# Interactive Rhythmic Cue Facilitates Gait Relearning in Patients with Parkinson's Disease

**DOI:** 10.1371/journal.pone.0072176

**Published:** 2013-09-30

**Authors:** Hirotaka Uchitomi, Leo Ota, Ken-ichiro Ogawa, Satoshi Orimo, Yoshihiro Miyake

**Affiliations:** 1 Department of Computational Intelligence and Systems Science, Tokyo Institute of Technology, Yokohama, Kanagawa, Japan; 2 Department of Neurology, Kanto Central Hospital, Setagaya, Tokyo, Japan; The Chinese University of Hong Kong, Hong Kong

## Abstract

To develop a method for cooperative human gait training, we investigated whether interactive rhythmic cues could improve the gait performance of Parkinson's disease patients. The interactive rhythmic cues ware generated based on the mutual entrainment between the patient's gait rhythms and the cue rhythms input to the patient while the patient walked. Previously, we found that the dynamic characteristics of stride interval fluctuation in Parkinson's disease patients were improved to a healthy 1/*f* fluctuation level using interactive rhythmic cues and that this effect was maintained in the short term. However, two problems remained in our previous study. First, it was not clear whether the key factor underpinning the effect was the mutual entrainment between the gait rhythms and the cue rhythms or the rhythmic cue fluctuation itself. Second, it was not clear whether or not the gait restoration was maintained longitudinally and was relearned after repeating the cue-based gait training. Thus, the present study clarified these issues using 32 patients who participated in a four-day experimental program. The patients were assigned randomly to one of four experimental groups with the following rhythmic cues: (a) interactive rhythmic cue, (b) fixed tempo cue, (c) 1/*f* fluctuating tempo cue, and (d) no cue. It has been reported that the 1/*f* fluctuation of stride interval in healthy gait is absent in Parkinson's disease patients. Therefore, we used this dynamic characteristic as an evaluation index to analyze gait relearning in the four different conditions. We observed a significant effect in condition (a) that the gait fluctuation of the patients gradually returned to a healthy 1/*f* fluctuation level, whereas this did not occur in the other conditions. This result suggests that the mutual entrainment can facilitate gait relearning effectively. It is expected that interactive rhythmic cues will be widely applicable in the fields of rehabilitation and assistive technology.

## Introduction

When two people walk together, the gait rhythms often synchronize naturally. This phenomenon is a typical example of interpersonal synchronization between human movements, which is often observed in daily life [1], [2]. The interpersonal synchronization has been examined using a dynamical systems approach [3] and is supported neurally by perception–action linkages [4] and mirror systems [5]. Also, studies of human gait control, which forms the basis of interpersonal synchronization, have investigated the interactions between central pattern generators (CPGs) in the spinal cord [6], [7] and the musculoskeletal system [8], [9], as well as the interlimb coordination patterns produced by the integration of multiple oscillator networks [10]. Moreover, functional brain imaging studies [11] and behavioral experiments [12] has investigated the dependency of cortical activation on the linkages between sensory inputs and motor outputs. However, the previous studies have focused on intrapersonal synchronization but have rarely focused on the mechanism that underpins interpersonal synchronization of gait rhythms beyond individual behavior. Specifically, it remains unclear the relationship between intrapersonal synchronization and interpersonal synchronization.

Our research group addresses the hypothesis that the mutual entrainment between human movements is an essential mechanism for the interpersonal synchronization. In the first stage, we modeled interpersonal synchronization via the mutual entrainment of gait rhythms between two humans based on this hypothesis [13], [14]. Then, based on the model, we developed a system, called “WalkMate”, to emulate the interpersonal synchronization of gait rhythms between two humans. The WalkMate system is a cross-feedback system to realize the cross-feedback loop between the gait rhythms generated by a human and the cue rhythms generated by the system itself based on the mutual entrainment [15]–[19]. More specifically, the WalkMate system provides the interactive rhythmic cues using nonlinear oscillators [19]. Pressure sensors and transmission devices are attached to the soles of the human's shoes, which detect the step timings while walking. The system obtains the step timings in sequence from the sensors, and calculates the stride intervals of the human in real time. The system generates rhythmic cues based on the oscillatory intervals of the oscillators. The system regulates the intervals to synchronize with the human's stride intervals. The rhythmic cues generated in this way, called “interactive rhythmic cues”, are provided from the WalkMate system to the human.

In the second stage, we investigated the dynamic characteristics of the intrapersonal gait dynamics of a human. We focused on Parkinson's disease (PD) as an example of our researches. PD is a neurodegenerative disease of the basal ganglia, and PD patients exhibit degraded repetitive movement and gait disturbances. The gait disturbances can manifest in various forms, including a festinating (i.e., accelerating) gait, a slow shuffling gait, or a highly variable stride interval [20]. From the viewpoint of the gait dynamics, healthy subjects exhibit 1/*f* fluctuation in their stride intervals [21], [22]. This means that healthy humans have a rhythm generator as an intrapersonal gait dynamics that generates gait rhythms with 1/*f* fluctuation. While on the other hand, the fractal scaling of the stride intervals of PD patients is reduced considerably from the 1/*f* fluctuation [23]. This means that the rhythm generator of the PD patients becomes dysfunctional and therefore can only generate gait rhythms with reduced-1/*f* fluctuation. Previously, we investigated the effects of human and WalkMate system cooperation [24]. The PD subjects in the experiment walked in response to rhythmic cues under three conditions (interactive condition, fixed tempo condition, and silent control condition) and we analyzed the dynamic characteristics of their stride intervals. The stride interval fluctuation of the PD subjects was boosted to a healthy 1/*f* fluctuation level due to only the interactive rhythmic cues generated by the WalkMate system and this characteristic fluctuation maintained after the cues ceased. These results suggest that the mutual entrainment between gait rhythms and cue rhythms helps to improve the intrapersonal gait performance of the PD subjects because the rhythm generator of the WalkMate system was based on mutual entrainment to gait rhythms of humans [19].

Here we focus on the fact that the interactive rhythmic cues generated by the WalkMate system fluctuates because the interactive rhythmic cues change depending on the gait rhythms generated by the PD subjects. The rhythm generator of the PD subjects, as the intrapersonal gait dynamics, generates gait rhythms with reduced-1/*f* fluctuation rather than healthy 1/*f* fluctuation. This means that the PD subjects received the interactive rhythmic cues with reduced-1/*f* fluctuation from the WalkMate system recursively by a cross-feedback loop through the WalkMate system. This suggests that the rhythm generator of the PD patients makes a functional recovery by the input of the interactive rhythmic cues with reduced-1/*f* fluctuation at least during the experiment. However, a previous study reported that the stride interval fluctuation of healthy elderly subjects approached a healthy 1/*f* fluctuation level when they walked while listening to 1/*f*-like fluctuating rhythmic cue, rather than fixed tempo rhythmic cue [25]. As in the case of our previous study, this report suggests that the rhythm generator of the healthy elderly subjects is recovered by the input of the 1/*f* fluctuating rhythmic cue. Hence, our previous study did not determine which was effective for improving the intrapersonal gait dynamics of the PD subjects, the interpersonal synchronization based on the interactive rhythmic cues or the cue fluctuation itself [24].

Furthermore, even though it was reported that the effect of gait performance improvement by the WalkMate system was maintained, this effect was only evaluated immediately after using the system. Therefore, it is unknown whether the WalkMate system facilitated gait relearning of the intrapersonal gait dynamics of the PD subjects for a while after using the system, which is important if this approach is to be used for gait rehabilitation. From a clinical viewpoint, it is not sufficient that the gait performance is restored only when the WalkMate system is used. Instead, the system is expected to recover the gait performance and the relearning of the intrapersonal gait dynamics for as long period as possible. The previous study did not address this issue. This issue is also important to investigate how the above-mentioned interpersonal synchronization influences to the intrapersonal gait dynamics of the PD subjects are established after using the WalkMate system.

Based on this background, the aim of the present study is to test the hypothesis that the interpersonal synchronization process based on the mutual entrainment of human gait rhythms and interactive cue rhythms, rather than the simple cue rhythms or the fluctuations in the cue rhythms, is effective for improving the gait performance of the PD patients and relearning of the intrapersonal gait dynamics. Note that we will use the phrase “interpersonal synchronization” in what follows even though synchronization is established between a human (PD subject) and the WakeMate system. The reason is that the WalkMate system is modeled on the gait dynamics of a human. To test this hypothesis, the effects of the interactive rhythmic cues generated by the WalkMate system, called “interactive WalkMate rhythmic cues”, on the gait performance improvement and the gait dynamics relearning are compared with fixed tempo rhythmic cue and 1/*f* fluctuating rhythmic cue during gait experiments.

## Materials and Methods

### Subjects

Thirty-two idiopathic PD patients (18 men and 14 women) participated in the experiment. The subjects had normal hearing and no dementia. The ages and disease durations of the PD subjects were 70.4±8.24 and 5.41±4.05 years (mean ± SD), respectively. PD has various representative severity indicators but the modified Hoehn and Yahr stage was 2.44±0.520. All the subjects were taking dopaminergic medication for the treatment of PD and were tested while “on” dopaminergic medication. Written informed consent was provided and the subjects were paid for participating. The Kanto Central Hospital Ethics Committee in Japan approved the following experimental procedures.

### Conditions and tasks

The 32 subjects were randomly assigned to four groups of eight. Each group participated in the four-day gait experiment program. Each of the four groups performed the experiment in a different condition: interactive WalkMate condition, fixed tempo condition, 1/*f* fluctuating tempo condition, or silent control condition. The interactive WalkMate condition provided interactive rhythmic cues, which was set for mutual entrainment with the subject's gait rhythm (interactive WalkMate rhythmic cue). The fixed tempo condition provided noninteractive, constant rhythmic cues, whose tempo was set to a subject's spontaneous gait rhythm (fixed tempo rhythmic cue). The 1/*f* fluctuating tempo condition provided noninteractive 1/*f* fluctuating rhythmic cues where the average of the tempo was set to the subject's spontaneous gait rhythm (1/*f* fluctuating tempo rhythmic cue). Here, the fluctuations in the cue sequence were derived from the time series of healthy young people's stride interval that showed 1/*f* fluctuation. The silent control condition provided no cue. In this between-subjects design, the eight subjects in each group received only one of the cue conditions. We measured one by one the PD subjects to ensure their random selection when forming the four groups. The PD subjects were recruited by a medical doctor and were grouped by an experimenter. All the subjects were unaware of the experimental contents, the procedures, and the conditions, except walking, until immediately before the experiments started.

**Figure 1:**
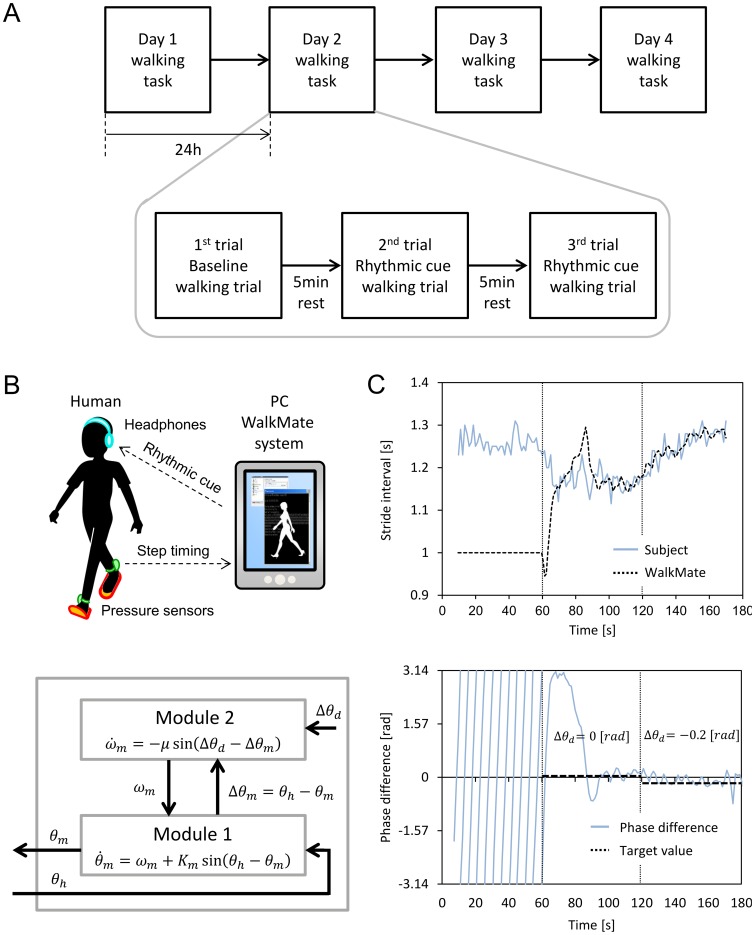
The experimental procedure and experimental system used to provide rhythmic cues. (A) The block diagram of the experimental procedure, which comprised a four-day gait experiment program. The daily walking task comprised one baseline walking trial and two rhythmic cue walking trials. In the baseline walking trial, the subjects walked alone without any rhythmic cues. In the rhythmic cue walking trials, they walked with rhythmic cues in different experimental conditions. (B) The schematic diagram showing the experimental system and the rhythm generator model implemented in the system. (C) The time course of changes in the stride interval and the step-to-cue phase difference of a healthy human walking with the system.

The experimental task occurred during four consecutive days from Tuesday to Friday (Figure 1A). Each subject started the experiment at the same time on each day (2:00 PM, 3:00 PM, or 4:00 PM). During days 1–3, the subjects performed three experimental trials on each day. The first trial was a baseline walking trial where the subjects walked alone without any cue (in the silent control condition) and the aim was to evaluate each subject's gait state on that day. The second and third trials were rhythmic cue walking trials where the subjects walked with rhythmic cue assistance in different experimental conditions, and the aims of these two trials were gait training with rhythmic cues in each experimental condition. The subjects were not instructed explicitly to synchronize their gait rhythms during rhythmic cues when the cues were provided in the rhythmic cue walking trial. On the day 4, the subjects performed the baseline walking trial only.

In all of the trials, the subjects walked along a corridor that measured 200 m in length. Each trial lasted for approximately 3 min and comprised 320 steps. The subjects wore headphones to listen to rhythmic cues and pressure sensors were attached to their shoes to collect gait rhythm information. When a foot was in the swing phase (the shoe was above the ground), the pressure sensor was in the “off” state. When a foot was in the stance phase (the shoe was on the ground), the pressure sensor was in the “on” state. A radio transmitter was connected to the pressure sensor, which sent the timestamp of the rising edge of the sensor's state to a laptop computer that collected the time series of step timings. In the fixed tempo condition, the cue rhythm remained constant throughout the trial, and interpersonal synchronization should be encouraged by setting the cue rhythms to the subject's spontaneous gait rhythms, rather than 10% faster, which has been used sometimes [26]. In the 1/*f* fluctuating tempo condition, the cue sequence was generated based on the fluctuation of the stride intervals of a healthy subject. In addition to the current experiment, we previously measured the time series data of the stride intervals of a healthy subject during normal gait performance and the stride intervals had 1/*f* fluctuation, which agreed with a previous report [23]. The intervals of the 1/*f* fluctuating tempo cues were based on the measured stride interval data, where only the mean was adjusted to the subject's spontaneous gait rhythms while the fluctuation was the same as that in the original data. In the interactive WalkMate condition, the cue rhythm changed in response to the subject's gait rhythms. The interactive cue rhythms were generated using the following system.

### Experimental system

The WalkMate system generates interactive rhythmic cues, which interact with the subject's gait rhythm and which is output for the subject [19]. The rhythm generator model of the WalkMate system has a hierarchical structure, as shown in Figure 1B. Module 1 is responsible for mutually synchronizing the subject's gait rhythms and cue rhythms via mutual entrainment. This process uses nonlinear oscillators [27], which have been shown to be effective for simulating CPGs [28]. Module 2 controls the step-to-cue phase difference between the sensory input from the pressure sensors and the output provided as rhythmic cues using a targeted phase difference value, i.e., the interpersonal synchronization between the subject's gait rhythms and cue rhythms. This provides feedback control, which determines the step-to-cue phase difference between the input and output of Module 1. The validity of this model is supported because human gait behaviors are governed hierarchically by spinal CPG-dependent rhythm modulation and via cerebellar and brainstem feedback control systems [29], [30]. This model is also supported by the dual process model [31] and a previous investigation of synchronized tapping [13], [14].

Next, we briefly describe the application of the WalkMate system [32]. Figure 1C shows examples of the stride intervals and phase difference for a healthy subject walking down a straight corridor with the WalkMate system. During the first 60 s, the subject walked alone without any rhythmic cue. The subject's stride intervals and the WalkMate's oscillator intervals were different and independent. During the next 60 s, cross-feedback occurred between the intervals of the subject and the WalkMate's oscillator where the target phase difference was set at 0 rad. The two time series approached each other via mutual entrainment and their phase difference converged in a stable manner to the target phase difference of 0 rad. During the last 60 s, the intervals of the subject and the WalkMate's oscillator were mutually synchronized with a target phase difference of 0.2 rad, which shows that there was a slight delay in the presentation of the rhythmic cues relative to the subject's steps. The subject's gaits then slowed and the WalkMate's oscillator intervals increased automatically with no noticeable change in the target phase difference. This showed that subject's stride intervals can be manipulated by controlling the target phase difference during interpersonal synchronization.

### Data analysis

Natural and biological temporal processes often have long-range correlations and fractal scaling. Long-range dependency, long memory, power laws, and 1/*f* fluctuation have been observed in time series from many domains [33], [34], and scaling laws are ubiquitous in physical, chemical, biological, cognitive, geological, social, and economic systems. Previous studies have reported the importance of evaluating the dynamic aspects of gait performance [23], [38]. It is possible to inspect the degree of scale invariance by plotting the fluctuations at different temporal resolutions. We quantified the long-range correlations using detrended fluctuation analysis (DFA) [23], [36], [37]. This technique has certain advantages compared with other methods (e.g., spectral or Hurst analyses) when dealing with nonstationary time series because it avoids the spurious detection of apparent long-range correlations that are artifacts of nonstationarity [36], [37] (see Supporting Information S1 for details of the method).

**Figure 2:**
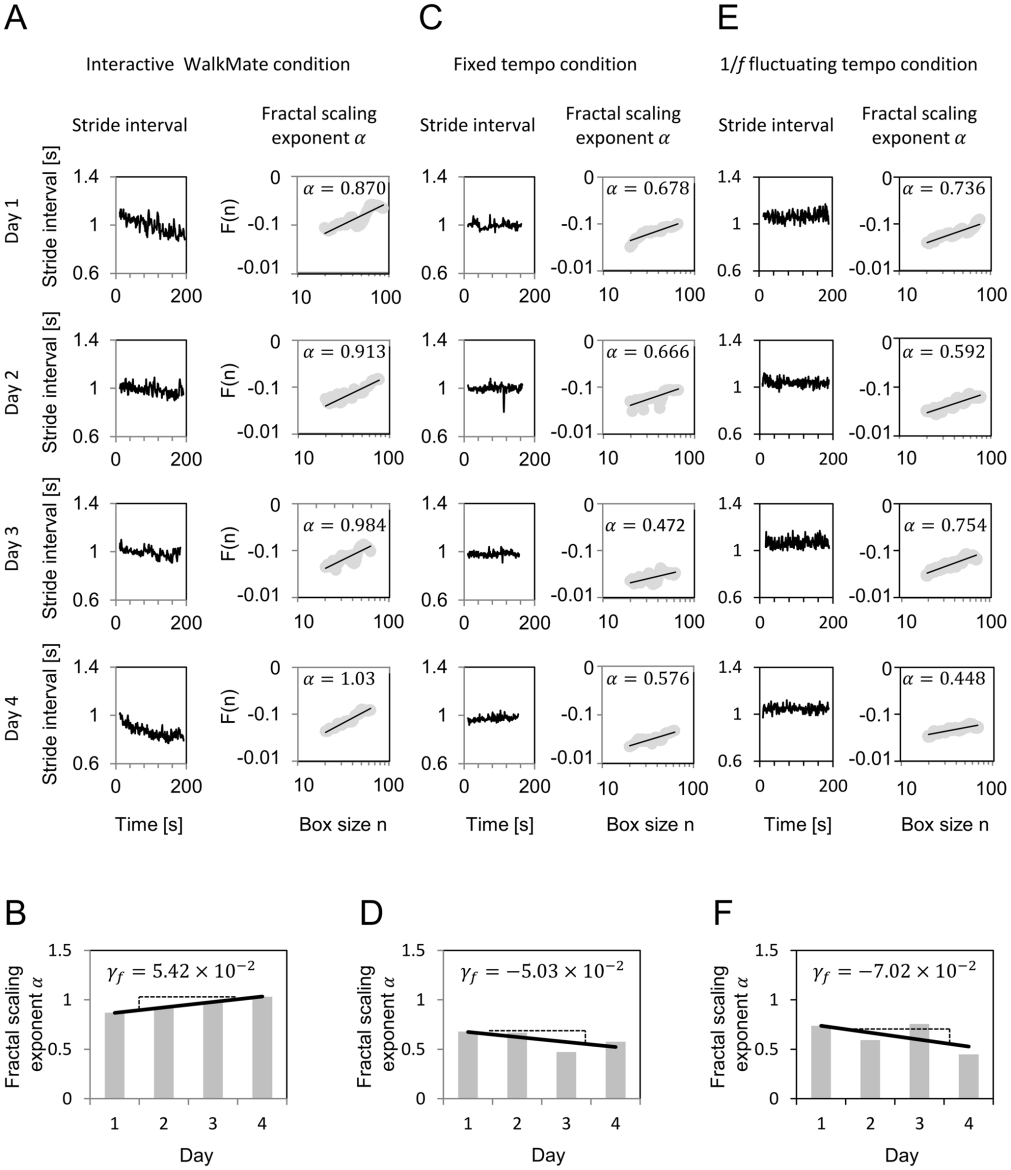
Example results of the gait relearning effects in Parkinson's disease subjects under three rhythmic cue conditions, i.e., one in the interactive WalkMate condition, another in the fixed tempo condition and the other in the 1/*f* fluctuating tempo condition. (A) The stride intervals and detrended fluctuation analysis (DFA) of the fractal scaling exponent *α* for the baseline walking trial of the subjects in the interactive WalkMate condition over four days. (B) The gait relearning effect *γ_f_* for the subjects in the WalkMate condition. In each four-day experiment, the fractal scaling exponent was calculated by applying the DFA to the stride interval time series and the fractal scaling exponents were evaluated using the linear regression slope of the gait relearning effect indicator *γ_f_*. The positive *γ_f_* value indicates a trend toward increased fractal scaling, which demonstrates the effect of interactive WalkMate rhythmic cues on gait relearning. (C) The stride intervals and DFA fractal scaling exponents *α* in the fixed tempo condition. (D) The gait relearning effect *γ_f_* in the fixed tempo condition. (E) The stride intervals and DFA fractal scaling exponents *α* in the 1/*f* fluctuating tempo condition. (F) The gait relearning effect *γ_f_* in the 1/*f* fluctuating tempo condition. The nonpositive *γ_f_* values indicate that there were no increasing trends in the fractal scaling exponents, which demonstrates that fixed tempo and 1/*f* fluctuating tempo rhythmic cues showed no gait relearning effects.

DFA yields a specific index *α*, which is related to the fractal scaling, and *α* provides a measure of structure of the signal in the time series of the original stride intervals (see Figure 2). Using DFA, the fractal scaling exponent *α*≈0.5 corresponds to rough and unpredictable white noise; *α*≈1.0 corresponds to 1/*f* fluctuation and long-range correlations; and *α*≈1.5 corresponds to a random walk process or Brownian noise [36]. Of the conventional gait parameters, the mean and coefficient of variation of the stride interval, which are related to the gait speed and gait variability, respectively, were also calculated to evaluate the data obtained from the PD subjects. The stride intervals of the right leg were analyzed. The first 20 strides and final five strides were not analyzed in each trial.

To quantify the gait relearning effect in the experiment, we evaluated the linear regression slopes *γ* for three gait parameters obtained during walking trials: DFA fractal scaling exponent *γ_f_*, mean *γ_m_*, and coefficient of variation *γ_c_* of the stride intervals. We defined the gait relearning effect *γ_f_*, *γ_m_*, and *γ_c_* as the slopes of each gait parameter in each day's baseline trials only. In the rhythmic cue walking trial, the interpersonal synchronization was also analyzed using circular statistical methods, including the Rayleigh test and circular variance (see [38] for the details of the circular methods). The Rayleigh test determines whether the population of input data is distributed uniformly around a circle. The circular variance indexes the variance of step-to-cue phase difference using a scale from 1 (no interpersonal synchronization between steps and cues, where the step-to-cue phase difference is distributed uniformly around the unit circle) to 0 (perfectly stable interpersonal synchronization with a unimodal distribution in the step-to-cue phase difference).

The descriptive statistics comprised the mean values and the standard deviations. Planned comparisons between the four experimental groups (interactive WalkMate group, fixed tempo group, 1/*f* fluctuating tempo group, and silent-control group) were analyzed using one-way factorial analyses of variance and subsequent tests used Fisher's least significant difference test. The statistical analyses were performed using SPSS® (SPSS Inc. Chicago, IL). One PD subject was absent from the gait experiment program on the second of the four days so the missing values were interpolated from the median of the data obtained from the same group and on the same day for the statistical analyses. The interpersonal synchronization results based on circular statistics were compared in the fixed tempo group, the 1/*f* fluctuating tempo group and the interactive WalkMate group using one-way factorial analyses of variance and subsequent tests used Fisher's least significant difference test. The circular statistical analyses were performed using MATLAB® (MathWorks, Natick, MA). The reported *p*-values were for two-sided tests and the level of significance was *p* = 0.05.

## Results

Before reviewing the statistical results, we discuss examples of the gait relearning effect for single subjects in the interactive WalkMate group, 1/*f* fluctuating tempo group, and fixed tempo group, as shown in Figure 2. Using each day's baseline walking trial, DFA was used to calculate the fractal scaling exponent *α* for the measured time series of the stride intervals of the subjects (Figure 2A). After computing the fractal scaling exponents for four days, the degree of the gait relearning effect *γ_f_* was evaluated using the linear regression slope based on these four values (Figure 2B). For the PD subjects in the interactive WalkMate group in Figure 2, the quantified gait relearning effect *γ_f_* was 5.42×10^–2^. The positive slope indicated a trend toward an increasing fractal scaling exponent, which suggests that the interactive WalkMate rhythmic cues boosted the subject's 1/*f* fluctuation over the course of the four days. Similarly, for the PD subjects in the fixed tempo group and 1/*f* fluctuating tempo group, the gait relearning effect *γ_f_* was evaluated after collecting the fractal scaling exponents for four days (Figures 2C and 2E). The gait relearning effect *γ_f_* in the fixed tempo group was –5.03×10^–2^ (Figure 2D), while the effect *γ_f_* in the 1/*f* fluctuating tempo group was –7.02×10^–2^ (Figure 2F). These results showed that there was no increasing trend in the fractal scaling exponent, which suggests that the experiments with the fixed tempo rhythmic cues or the 1/*f* fluctuating tempo rhythmic cues did not improve the stride interval fluctuations in these subjects.

### Baseline gait performance of the PD subjects

The baseline walking trial on the first day of the experiment was the same for the interactive WalkMate group, fixed tempo group, 1/*f* fluctuating tempo group, and silent control groups in terms of the fractal scaling exponent, the mean, and the coefficient of variation for stride intervals, i.e., *F*(3,28)  = 0.0424, *F*(3,28)  = 0.367, *F*(3,28)  = 0.366, respectively, and *p*>0.1 in all cases (Table 1). Thus, the evaluation indicators showed that all of the groups had the same gait performance states before the experimental program commenced.

**Table 1:** Initial states of gait parameters in each group of Parkinson's disease (PD) subjects.

	Group of Parkinson's disease's subjects				*F*-test
Stride Interval	Silent control group	Fixed tempo group	1/*f* Fluctuating tempo group	Interactive WalkMate group	*p*-value
Fractal scaling exponent	0.759	0.769	0.685	0.735	0.7
Mean [s]	1.03	1.04	1.06	1.01	0.8
Coefficient of variation	0.0309	0.0299	0.0311	0.0353	0.8

### Gait relearning effect in fractal scaling of stride intervals

**Figure 3:**
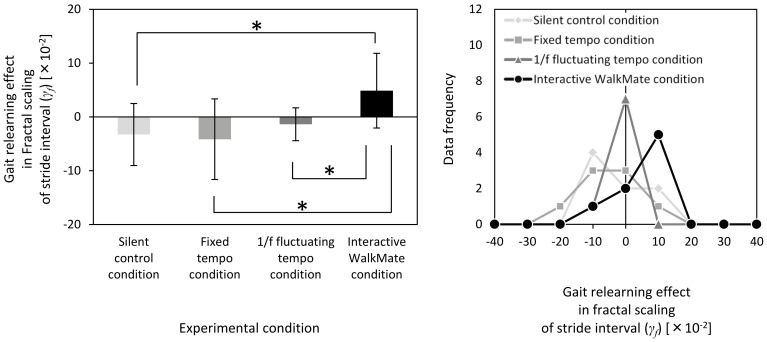
Statistical results and distributions of the data frequencies for the gait relearning effects in the fractal scaling *γ_f_* of the stride intervals for the silent control, fixed tempo, 1/*f* fluctuating tempo, and interactive WalkMate conditions. Only the interactive WalkMate condition exhibited an increased fractal scaling exponent trend throughout the trial period, which is demonstrated by the positive value. This indicates that the gait experiment program using interactive WalkMate rhythmic cues boosted the fractal scaling of the stride interval fluctuation of the PD subjects to a healthy 1/*f* level. The asterisk in the figure indicates a significant difference at *p*<0.05.

Figure 3 summarizes the gait relearning effect *γ_f_* in all four experimental groups. The experimental conditions affected *γ_f_* the fractal scaling of stride intervals over the four-day program, i.e., *F*(3,28)  = 3.55, *p*<0.05 (Figure 3). The interactive WalkMate group (*γ_f_* = 4.87×10^–2^±6.94×10^–2^) had a significantly larger *γ_f_* compared with the unassisted silent control group (*γ_f_*  = –3.27×10^–2^±5.76×10^–2^), the fixed tempo group (*γ_f_* = –4.15×10^–2^±7.50×10^–2^), and the 1/*f* fluctuating tempo group (*γ_f_* = –1.37×10^–2^±3.07×10^–2^) (*p*<0.05 in all cases). There were no differences between the silent control group, the fixed tempo group, and the 1/*f* fluctuating tempo group (*p*>0.05 in all cases).

### Gait relearning effect on the mean and the coefficient of variation for stride intervals

**Figure 4:**
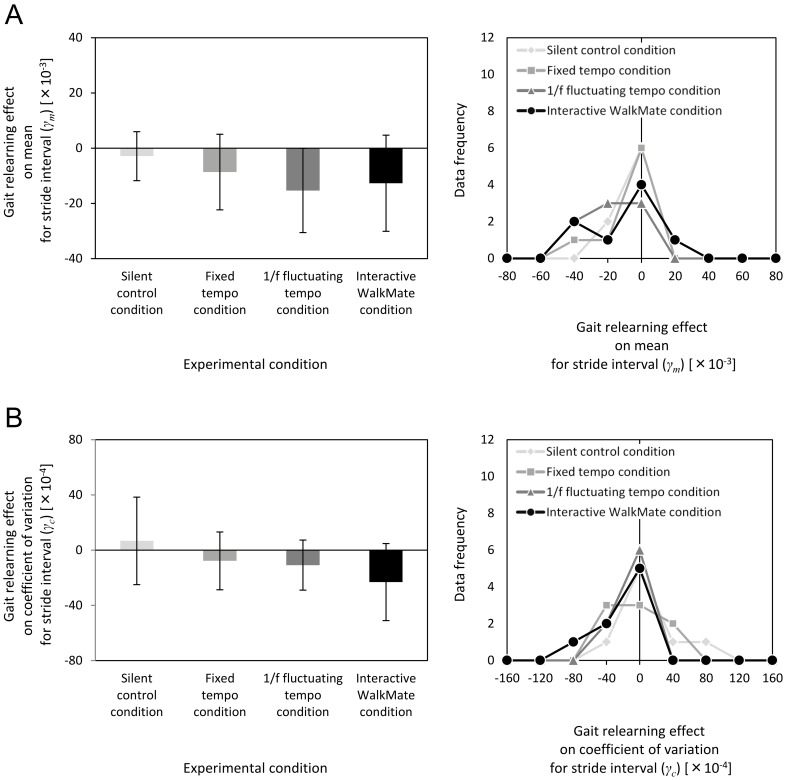
Statistical results and distributions of the data frequencies for the gait relearning effects on the mean *γ_m_*, and the coefficient of variation *γ_c_*, of the stride interval for the silent control, fixed tempo, 1/*f* fluctuating tempo, and interactive WalkMate conditions. (A) Experimental results showing the gait relearning effects on the mean stride interval *γ_m_*. (B) Experimental results showing the gait relearning effects on the coefficient of variation *γ_c_* of the stride interval.

Figure 4 summarizes the gait relearning effects *γ_m_* and *γ_c_* for all four experimental groups. The gait relearning effect did not differ significantly between the four experimental groups in terms of the mean stride interval *γ_m_*, i.e., *F*(3,28)  = 1.17, *p* = 0.3 (Figure 4A). For the silent control group, *γ_m_* = –2.89×10^–3^±8.90×10^–3^; for the fixed tempo group, *γ_m_* = –8.65×10^–3^±13.7×10^–3^; for the 1/*f* fluctuating tempo group, *γ_m_* = –15.3×10^–3^±15.3×10^–3^; and for the interactive WalkMate group, *γ_m_* = –12.7×10^–3^±17.4×10^–3^. Similarly, the gait relearning effect did not differ significantly between the three experimental groups in terms of the coefficient of variation *γ_c_* for stride intervals, i.e., *F*(3,28)  = 1.90, *p* = 0.2 (Figure 4B). For the silent control group, *γ_c_* = 6.72×10^–4^±31.7×10^–4^; for the fixed tempo group, *γ_c_* = –7.75×10^–4^±20.9×10^–4^; for the 1/*f* fluctuating tempo group, *γ_c_* = –10.9×10^–4^±18.1×10^–4^; and for the interactive WalkMate group, *γ_c_* = –23.2×10^–4^±27.9×10^–4^.

### Interpersonal synchronization between steps and cues

To explore the relationship between the gait relearning effects and the degree of interpersonal synchronization between gait rhythms and cue rhythms, the step-to-cue phase differences were analyzed during the rhythmic cue walking trials for the fixed tempo group, the 1/*f* fluctuating tempo group, and the interactive WalkMate group. The results of the Rayleigh test showed that the stride intervals of the PD subjects in the fixed tempo group and the 1/*f* fluctuating tempo group did not synchronize with the rhythmic cue (Rayleigh test, *p*>0.1). By contrast, the stride intervals of the PD subjects in the interactive WalkMate group were synchronized with the rhythmic cues (Rayleigh test, *p*<0.01). Similarly, the index of the stability of interpersonal synchronization showed that the experimental conditions affected the mean of the circular variance, *F*(2,141)  = 6.87×10^–3^, *p*<0.01. The mean of the circular variance in the interactive WalkMate group (mean ± SD = 0.0233±0.0315) was lower than that in the fixed tempo group (mean ± SD = 0.982±0.0120) and the 1/*f* fluctuating tempo group (mean ± SD = 0.946±0.0711) (*p*<0.01 in both cases).

## Discussion

The present study investigated the effect of interpersonal synchronization between human gait rhythms and interactive WalkMate rhythmic cues on the relearning of intrapersonal gait dynamics. The experimental results confirmed our hypothesis that the interpersonal synchronization process promoted the improvement of intrapersonal gait relearning. Thus, interpersonal synchronization based on the mutual entrainment between human gait rhythms and interactive rhythmic cues was the mechanism underpinning the relearning of intrapersonal gait dynamics, rather than the simple cue sequence or cue fluctuations.

In our experiments, the gait relearning effect *γ_f_* of the fractal scaling exponent of the stride interval was significantly higher in the interactive WalkMate group than the fixed tempo group, the 1/*f* fluctuating tempo group, and the silent control group. The positive value of *γ_f_* in the interactive WalkMate group demonstrated that there was an increasing trend in the fractal scaling exponent, which suggests that there was a gain in 1/*f* fluctuation of the stride intervals of the PD subjects over the course of four days. By contrast, the nonpositive values of *γ_f_* in the other groups demonstrated the absence of this trend, which suggests that the stride interval fluctuations of the PD subjects were not improved in these groups.

Previous studies have shown that the stride interval fluctuations of healthy subjects have 1/*f* fluctuation and a high fractal scaling exponent [21], [22]. By contrast, the stride interval fluctuations of the PD subjects have reduced-1/*f* fluctuation and a lower fractal scaling exponent compared with healthy subjects [23]. In our experiments, the stride interval fluctuations of the PD subjects tended to approach a healthy 1/*f* fluctuation level from a reduced-1/*f* fluctuation level. Therefore, we conclude that the interpersonal synchronization of interactive WalkMate rhythmic cues with human gait rhythms facilitated the relearning of intrapersonal gait dynamics in the PD subjects, from the perspective of the stride interval fluctuation.

The present study also showed that *γ_f_* in the fixed tempo condition and the 1/*f* fluctuating tempo condition did not differ significantly from that in the silent control condition. The result suggests that the interpersonal synchronization between gait rhythms and cue rhythms based on the mutual entrainment facilitated the recovery of 1/*f* fluctuation in the stride intervals of the PD subjects. If the PD subjects in the interactive WalkMate condition, the fixed tempo condition, and the 1/*f* fluctuating tempo condition had exhibited an intrapersonal gait relearning effect, we could have concluded that the simple cue sequence contributed to the recovery of 1/*f* fluctuation in stride intervals. If the PD subjects in the interactive WalkMate condition and the 1/*f* fluctuating tempo condition (but not the fixed tempo condition) had exhibited the intrapersonal gait relearning effect, we could have concluded that the fluctuation in the cue sequence contributed to the recovery of 1/*f* fluctuation in stride intervals.

However, only the interactive WalkMate condition exhibited the intrapersonal gait relearning effect in our experiments. The only difference between the interactive WalkMate rhythmic cues and the other cues was the presence of the interpersonal synchronization process. Thus, the mutual entrainment is considered to be effective for relearning the healthy gait dynamics. The present study suggested that the mutual entrainment of gait rhythms and cue rhythms facilitates the intrapersonal gait restoration and relearning, which indicates the potential efficacy of the WalkMate system in health preservation training programs and motor rehabilitation focused on gait performance.

Thus, the WalkMate system facilitated the gait restoration and relearning in the PD subjects, but what aspects of the PD subjects' gait dynamics did it affect? PD involves basal ganglia disease [20]. It is known that the basal ganglia connect the supplementary motor cortex (SMC) with sensory areas including the auditory area. It has been suggested that the activation of the basal ganglia and the SMC has a role in the generation of intrapersonal rhythms related to the planning of movement sequences [12]. It has been reported that the SMC is not activated when external cues are provided to PD patients [11]. This suggests that the external cues inhibit the function of the SMC during the neural information processing of movement sequences. By contrast, we observed intrapersonal gait restoration and relearning in the PD subjects in our experiments. The results suggest that the interactive rhythmic cues generated by the WalkMate system could not be regarded as external cues by the SMC. It is therefore suggested that the cross-feedback loop, formed between the system and the PD subjects recursively based on the mutual entrainment, could compensate for and increase the audio-motor linkage neural connection to control intrapersonal gait rhythms related to the gait sequence planning. Thus, we consider that the WalkMate system helps to activate and maintain the neural pathway. In addition, previous studies have suggested that the timing systems of humans and other living organisms entrain their internal rhythms to external rhythmic events [39]–[41]. In the human timing system, the basal ganglia and the SMC synchronize internal rhythms with external rhythmic events and retain the modified internal rhythms after the events [42], [43]. In our experiments, the step-to-cue phase difference suggested that the subjects' steps were well synchronized with the interactive WalkMate rhythmic cues. Our experimental results suggest that the interpersonal synchronization between the PD subjects and WalkMate system activated the human timing system temporarily and helped to restore the PD subject's gait performance to a healthy condition in terms of the intrapersonal gait dynamics.

Previous studies have indicated that interactions between multiscale components are key factors in the emergence of 1/*f* fluctuation [22], [33]–[37], [44]–[47]. It has been reported, for instance, that the time series of the inter-tap intervals when a human taps a finger freely has 1/*f* fluctuation [48], [49]. If two humans tap their fingers in alternating sequences, the time series of the inter-tap intervals also has 1/*f* fluctuation [50]. In the former case, intrapersonal interactions between various multiscale components within a human generate rhythm dynamics with 1/*f* fluctuation, whereas in the latter case, interpersonal interactions between two humans generate rhythm dynamics with 1/*f* fluctuation. Specifically, the latter case is a good example of the multiscale coupling of intrapersonal rhythm-generating dynamics and interpersonal rhythm-generating dynamics via a cross-feedback loop. It is considered that this multiscale coupling generates 1/*f* fluctuation. Hence, it generally appears that supporting the multiscale interactions associated with human movements can compensate for disordered human motor functions directly but it also helps to activate the recovery of the original ability in humans.

It should be noted that the gait performance has kinematic characteristics as well as dynamic characteristics. From the viewpoint of gait dynamics, previous studies have reported that the stride interval fluctuation of PD patients often exhibited reduced-1/*f* fluctuation rather than 1/*f* fluctuation [23]. It has also been suggested that 1/*f* fluctuation is not correlated with the mean and variability of stride intervals [24]. In our study, the mean *γ_m_* and coefficient of variation *γ_c_* of stride intervals did not differ significantly between all the four conditions when we analyzed the intrapersonal gait relearning effect. By contrast, only the interactive WalkMate condition had a significantly different fractal scaling exponent *γ_f_* compared with the fixed tempo condition, the 1/*f* fluctuating tempo condition, and the silent control condition. These results suggest that the interactive WalkMate rhythmic cues produced the intrapersonal gait relearning effect. Thus, it is likely that there are several stages during the recovery of walking where the dynamic characteristics of the gait performance make a transition to a healthy state before the kinematic characteristics of the intrapersonal gait performance are improved.

A previous study conducted gait rehabilitation, in which a three-week home rhythmic cue program using fixed tempo rhythmic cues improved the gait speed and balance [51]. Our experimental results showed no significant effects on the intrapersonal gait relearning effect in terms of the mean and the coefficient of variation of stride intervals during the four-day experimental program. Therefore, a longer training period may be necessary to quantify the effects of the WalkMate system on the kinematic characteristics, such as the speed or variation in the intrapersonal gait performance. Thus, further research is required to investigate this issue.

The limitations of the present study should also be noted. First, thirty-two PD subjects participated in the experiment and four experimental groups were used to compare the effects of the four experimental conditions. Thus, there were eight PD subjects in each group, which is a relatively small sample size for a clinical trial. Second, the present study focused on the effects on the intrapersonal gait dynamics of the interactive rhythmic cue generated by the WalkMate system and noninteractive cues. However, various interactions between steps and cues are possible, whereas our experiment considered only one case. Therefore, other interactive rhythmic cues should be studied to determine their effects on the intrapersonal gait relearning.

## Conclusions

In summary, the stride interval fluctuation of the PD subjects had a low fractal scaling at baseline, which indicated gait impairment [23], [37]. The PD subjects were subjected to gait training with interactive rhythmic cues for four consecutive days, and their fractal scaling showed an increasing trend over the four days. This suggests that the interaction between gait rhythms and cue rhythms helped the PD subjects to relearn their intrapersonal gait dynamics and they approached a healthy 1/*f* fluctuation level.

The experimental results confirmed our hypothesis that the mutual entrainment was effective for relearning the intrapersonal gait dynamics of the PD subjects. Our previous study showed that the impaired gait stability and dynamics of PD patients could be improved when walking with the WalkMate system [18], [24]. This is a good example of the coupling of intrapersonal and interpersonal dynamical systems via a cross-feedback loop [27], [47], and the present study provided a positive evidence of the benefits of human-machine interactions during rehabilitation.

Future research should investigate the effects of interactive WalkMate rhythmic cues on gait relearning using more subjects and for a longer period to provide the evidence that is more convincing. It would also be useful to test social implementations of this human-machine interaction during home-based and community-based rehabilitation. The interactive WalkMate rhythmic cue can be adopted as a rehabilitation method for promoting effective gait relearning. This noninvasive, flexible, portable, and low-cost therapeutic intervention may improve the mobility, stability, and quality of life of PD patients.

## Supporting Information

Supporting Information S1 (DOCX)

## References

[1] Zivotofsky AZ, Hausdorff JM (2007) The sensory feedback mechanisms enabling couples to walk synchronously: an initial investigation. Journal of Neuroengineering and Rehabilitation 4 (28): 1–5. 10.1186/1743-0003-4-28 PMC1973071 17686150

[2] Strogatz S (2003) SYNC: The emerging science of spontaneous order. New York: Theia Books. 338 p.

[3] Schmidt RC, Carello C, Turvey MT (1990) Phase transitions and critical fluctuations in the visual coordination of rhythmic movements between people. J Exp Psychol Hum Percept Perform 16: 227–247. 2142196 10.1037//0096-1523.16.2.227

[4] Hommel B, Müsseler J, Aschersleben G, Prinz W (2001) The theory of event coding (TEC): a framework for perception and action planning. Behav Brain Sci 24: 849–870. 12239891 10.1017/s0140525x01000103

[5] Rizzolatti G, Fadiga L, Gallese V, Fogassi L (1996) Premotor cortex and the recognition of motor actions. Brain Res Cogn Brain Res 3: 131–141. 8713554 10.1016/0926-6410(95)00038-0

[6] Grillner S (1985) Neurobiological bases of rhythmic motor acts in vertebrates. Science 228: 143–149. 3975635 10.1126/science.3975635

[7] Kopell N, Ermentrout GB (1988) Coupled oscillators and the design of central pattern generators. Math Biosciences 90: 87–109.

[8] Taga G, Yamaguchi Y, Shimizu H (1991) Self-organized control of bipedal locomotion by neural oscillators in unpredictable environment. Biol Cybern 65: 147–159. 1912008 10.1007/BF00198086

[9] Taga G (1995) A model of the neuro-musculo-skeletal system for human locomotion. I. Emergence of basic gait. Biol Cybern 73: 97–111. 7662771 10.1007/BF00204048

[10] Yuasa H, Ito M (1990) Coordination of many oscillators and generation of locomotory patterns. Biol Cybern 63: 177–184.

[11] Cunnington R, Iansek R, Bradshaw JL, Phillips JG (1995) Movement-related potentials in Parkinson's disease. Presence and predictability of temporal and spatial cues. Brain 118 (Pt 4): 935–950. 10.1093/brain/118.4.935 7655889

[12] Georgiou N, Bradshaw JL, Iansek R, Phillips JG, Mattingley JB, et al (1994) Reduction in external cues and movement sequencing in Parkinson's disease. J Neurol Neurosurg Psychiatry 57: 368–370. 8158189 10.1136/jnnp.57.3.368 PMC1072831

[13] Miyake Y, Onishi Y, Pöppel E (2004) Two types of anticipation in synchronous tapping. Acta Neurobiol Exp (Wars) 64: 415–426. 15283483 10.55782/ane-2004-1524

[14] Takano K, Miyake Y (2007) Two types of phase correction mechanism involved in synchronized tapping. Neurosci Lett 417: 196–200. 17395371 10.1016/j.neulet.2007.02.044

[15] Miyake Y, Shimizu H (1994) Mutual entrainment based human-robot communication field. Proceeding of 3rd IEEE International Workshop on Robot and Human Communication (ROMAN '94) 118–123.

[16] Miyake Y, Tanaka J (1997) Mutual-entrainment-based internal control in adaptive process of human-robot cooperative walk. Proceeding of IEEE International Conference on Systems, Man, and Cybernetics 1: 293–298.

[17] Miyake Y, Miyagawa T, Tamura Y (2002) Man-machine interaction as co-emergence communication. Transactions of the Society of Instrument and Control Engineers E-2: 195–206.

[18] Muto T, Herzberger B, Hermsdoerfer J, Pöppel E, Miyake Y (2007) Virtual robot for interactive gait training: improving regularity and dynamic stability of the stride patterns. IEEE/ICME International Conference on Complex Medical Engineering 1240–1247.

[19] Miyake Y (2009) Interpersonal synchronization of body motion and the Walk-Mate walking support robot. Robot IEEE Trans 25: 638–644.

[20] Jankovic JJ, Tolosa E, editors (2006) Parkinson's disease and movement disorders. 5th ed. Philadelphia: Lippincott, Williams & Wilkins. 740 p.

[21] Scafetta N, Marchi D, West BJ (2009) Understanding the complexity of human gait dynamics. Chaos 19: 026108 doi: 10.1063/1.3143035 19566268 10.1063/1.3143035

[22] Hausdorff JM, Purdon PL, Peng CK, Ladin Z, Wei JY, et al (1996) Fractal dynamics of human gait: stability of long-range correlations in stride interval fluctuation. J Appl Physiol 80: 1448–1457. 8727526 10.1152/jappl.1996.80.5.1448

[23] Hausdorff JM (2009) Gait dynamics in Parkinson's disease: common and distinct behavior among stride length, gait variability, and fractal-like scaling. Chaos 19: 026113–026111–026114. 19566273 10.1063/1.3147408 PMC2719464

[24] Hove MJ, Suzuki K, Uchitomi H, Orimo S, Miyake Y (2012) Interactive rhythmic auditory stimulation reinstates natural 1/*f* timing in gait of Parkinson's patients. PLoS ONE 7: e32600. 22396783 10.1371/journal.pone.0032600 PMC3292577

[25] Kaipust JP, McGrath D, Mukherjee M, Stergiou N (2012) Gait variability is altered in older adults when listening to auditory stimuli with differing temporal structures. Ann Biomed Eng 40: 2507–2509. 10.1007/s10439-012-0654-9 22956164

[26] Thaut MH, Abiru M (2010) Rhythmic auditory stimulation in rehabilitation of movement disorders: a review of the current research. Music Perception 27: 263–269.

[27] Kuramoto Y (1984) Chemical oscillations, waves, and turbulence. New York: Springer-Verlag.

[28] Kopell N, Ermentrout GB (1988) Coupled oscillators and the design of central pattern generators. Math Biosci 90: 87–109.

[29] Orlovsky GN, Deliagina TG, Grillner S (1999) Neural control of locomotion. From mollusk to man. New York: Oxford University Press. 322 p.

[30] Shumway-Cook A, Woollacott M (1995) Motor control: theory and practical applications. Lippincott, Williams & Wilkins. 475 p.

[31] Mates J (1994) A model of synchronization of motor acts to a stimulus sequence. I. Timing and error corrections. Biol Cybern 70: 463–473. 8186306 10.1007/BF00203239

[32] Uchitomi H, Suzuki K, Hove MJ, Orimo S, Wada Y, et al.. (2012) Gait rhythm of Parkinson's disease patients and an interpersonal synchrony emulation system based on cooperative gait. In: Wu JL, Ito K, Tobimatsu S, Nishida T, Fukuyama H, editors. Biomedical engineering and cognitive neuroscience for healthcare: interdisciplinary applications, Tokyo: Springer-Verlag. 38–53.

[33] Newman MEJ (2005) Power laws, Pareto distributions, and Zipf's law. Contemporary Physics 46: 323–351.

[34] Kello CT, Brown GDA, Ferrer-i-Cancho R, Holden JG, Linkenkaer-Hansen K, et al (2010) Scaling laws in cognitive sciences. Trends Cogniti Sci 14: 223–232. 10.1016/j.tics.2010.02.005 20363176

[35] Hausdorff JM (2007) Gait dynamics, fractals and falls: finding meaning in the stride-to-stride fluctuations of human walking. Hum Mov Sci 26: 555–589. 17618701 10.1016/j.humov.2007.05.003 PMC2267927

[36] Peng C-K, Havlin S, Stanley HE, Goldberger AL (1995) Quantification of scaling exponents and crossover phenomena in nonstationary heartbeat time series. Chaos 5: 82–87. 11538314 10.1063/1.166141

[37] Goldberger AL, Amaral LA, Hausdorff JM, Ivanov PC, Peng CK, et al (2002) Fractal dynamics in physiology: alterations with disease and aging. Proc Natl Acad Sci 99: 2466–2472. 11875196 10.1073/pnas.012579499 PMC128562

[38] Fisher NI (1993) Statistical analysis of circular data. Cambridge: Cambridge University Press. 277 p.

[39] Hasegawa A, Okanoya K, Hasegawa T, Seki Y (2011) Rhythmic synchronization tapping to an audio–visual metronome in budgerigars. Sci Rep 1: 120. 22355637 10.1038/srep00120 PMC3216601

[40] Schwartze M, Keller PE, Patel AD, Kotz SA (2011) The impact of basal ganglia lesions on sensorimotor synchronization, spontaneous motor tempo, and the detection of tempo changes. Behavioural Brain Research 216: 685–691. 20883725 10.1016/j.bbr.2010.09.015

[41] Graybiel A, Aosaki T, Flaherty A, Kimura M (1994) The basal ganglia and adaptive motor control. Science 265: 1826–1831. 8091209 10.1126/science.8091209

[42] Kotz SA, Schwartze M, Schmidt-Kassow M (2009) Non-motor basal ganglia functions: a review and proposal for a model of sensory predictability in auditory language perception. Cortex 45: 982–990. 19361785 10.1016/j.cortex.2009.02.010

[43] Grahn JA, Brett M (2009) Impairment of beat-based rhythm discrimination in Parkinson's disease. Cortex 45: 54–61. 19027895 10.1016/j.cortex.2008.01.005

[44] Hausdorff JM, Lertratanakul A, Cudkowicz ME, Peterson AL, Kaliton D, et al (2000) Dynamic markers of altered gait rhythm in amyotrophic lateral sclerosis. J Appl Physiol 88: 2045–2053. 10846017 10.1152/jappl.2000.88.6.2045

[45] Bartsch R, Plotnik M, Kantelhardt JW, Havlin S, Giladi N, et al (2007) Fluctuation and synchronization of gait intervals and gait force profiles distinguish stages of Parkinson's disease. Physica A 383: 455–465. 18163154 10.1016/j.physa.2007.04.120 PMC2156195

[46] Delignieres D, Torre K (2009) Fractal dynamics of human gait: a reassessment of the 1996 data of Hausdorff et al. J Appl Physiol 106: 1272–1279. 19228991 10.1152/japplphysiol.90757.2008

[47] Herman T, Giladi N, Gurevich T, Hausdorff JM (2005) Gait instability and fractal dynamics of older adults with a “cautious” gait: why do certain older adults walk fearfully? Gait Posture 21: 178–185. 15639397 10.1016/j.gaitpost.2004.01.014

[48] Gilden DL, Thornton T, Mallon MW (1995) 1/*f* noise in human cognition. Science 267: 1837–1839. 7892611 10.1126/science.7892611

[49] Thornton TL, Gilden DL (2005) Provenance of correlations in psychological data. Psychonomic Bull Rev 12: 409–441. 10.3758/bf03193785 16235626

[50] Takenaka T, Ogata T, Ueda K (2006) Temporal co-creation between self and others with multi-sensory inputs. Adv Eng Informat 20: 321–333.

[51] Nieuwboer A, Kwakkel G, Rochester L, Jones D, van Wegen E, et al (2007) Cueing training in the home improves gait-related mobility in Parkinson's disease: the RESCUE trial. J Neurol Neurosurg Psychiatry 78: 134–140. 17229744 10.1136/jnnp.200X.097923 PMC2077658

