# Supplementary material for: Interactive Rhythmic Cue Facilitates Gait Relearning in Patients with Parkinson's Disease
Source: PLoS One. 2013 Sep 30;8(9):e72176. doi: 10.1371/journal.pone.0072176 (PMC3787045; doi:10.1371/journal.pone.0072176)
Supplement: Supporting Information S1 — (DOCX) [file pone.0072176.s001.docx]

**Supporting Information S1**

**for “Interactive Rhythmic Cue Facilitates Gait Relearning**

**in Patients with Parkinson’s Disease”**

Hirotaka Uchitomi^1^, Leo Ota^1^, Ken-ichiro Ogawa^1^, Satoshi Orimo^2^, and Yoshihiro Miyake^1^

^1^Department of Computational Intelligence and Systems Science, Tokyo Institute of Technology, Yokohama, Kanagawa, Japan,

^2^Department of Neurology, Kanto Central Hospital, Setagaya, Tokyo, Japan

**Contents**

1. Supplementary Methods ………………………………………………………….2
   1. Rhythm Generator Model implemented in the WalkMate System …………2
   2. Gait Data …………………………………………………….......................3
   3. Data Analysis ……………………………………………………………….4

# 1 Supplementary Methods

## 1.1 Rhythm Generator Model implemented in the WalkMate System

The information of gait rhythms was collected using pressure sensors attached on the shoes of each subject, which was relayed to a laptop via radio frequency every 10 ms. The collected time series of the gait rhythms was processed in real time to calculate the stride intervals. The stride intervals were not only saved but also were used in generating cue rhythms. In the rhythmic cue walking trials, the first rhythmic cue started after 20 s of walking. The subject’s stride intervals during this initial stage determined the initial interval of the rhythmic cues (based on the mean of five stride intervals, after excluding extreme values). The cue rhythms remained constant throughout the trial in the fixed tempo cue condition. To encourage synchronization, the cue rhythms were set to the subject’s spontaneous stride intervals, rather than 10% faster, which has been used in other studies　[26]. In the 1/*f* fluctuating tempo condition, the cue rhythms showed 1/*f* fluctuation. The fluctuation was derived from the stride intervals of healthy humans with 1/*f* fluctuation. The average intervals of the cue rhythms were set to the subject’s spontaneous stride intervals as same as the fixed tempo cue condition. In the interactive WalkMate condition, the cue rhythms changed in response to the subject’s stride intervals.

The rhythm generator model implemented in the WalkMate system is a computer algorithm for controlling the cue rhythms ran in an Open Linux SUSE environment on a Panasonic CF-W5 laptop computer. The rhythm generator model is composed of two modules hierarchically. Module 1 is to mutually entrain the rhythms of the WalkMate’s oscillator into the subject’s gait rhythms. Module 2 is to adjust the step-to-cue phase difference between the system and the subject to a given target phase difference.

Module 1 is described as

 (1)

where *θ_m_* represents the phase of the WalkMate’s oscillator and *ω_m_* denotes the frequency of the WalkMate’s oscillator. When *θ_m_* is an integer multiple of 2*π*, the system transmits a cue to the subject. The input variable *θ_h_* of this equation represents the phase of the subject’s gait rhythms, which is estimated based on the stride intervals. *K_m_* (>0) designates a given coupling constant.

Module 2 is described as

 (2)

where *Δθ_m_* (= *θ_h_* − *θ_m_*), *Δθ_d_*, and *μ* denote the step-to-cue phase difference, the target phase difference, and the control gain, respectively. Model 2 is responsible for adjusting the step-to-cue phase difference *Δθ_m_* to the target value *Δθ_d_*. These equations can be applied to both the right and left legs using a phase shift of *π*. In this study, the values of *K_m_*, *μ*, and *Δθ_d_* are set to 0.5, 0.32, and 0.2, respectively.

The rhythm generator model is appropriate because it has been shown that human gait behaviors are governed hierarchically by spinal CPG-dependent rhythm modulation and via cerebellar and brainstem feedback control systems [28–30]. Our approach is also supported by the dual process model [31] and the results of our synchronization tapping experiments [13,14].

## 1.2 Gait Data

We use the time series of the stride intervals of each subject to evaluate their gaits. The subject’s stride interval *T_h_* is described as

$T_{h}(i)=t_{h}(i+1)-t_{h}(i)$ (3)

where *T_h_* means the difference between *t_h_*(*i*+1) and *t_h_*(*i*) with the same leg. Here, *t_h_*(*i*) is the *i*-th step timing of the subject. This equation is also applicable to rhythmic cues, after replacing the suffix *h* (for human) with *m* (for cue).

In the present study, the phase difference *Δθ_h_*(*i*) for the *i*-th step is determined based on the difference between *t_h_*(*i*) and *t_m_*(*i*), as shown in

$\Delta\theta_{h}(i)=(t_{h}(i)-t_{m}(i))\frac{2\pi}{T_{h}(i)}$ (4)

## 1.3 Data Analysis

We provide a brief description of the DFA algorithm, which has been used in previous studies [30,36]. (a) Starting with a correlated signal *u*(*i*), where *i* = 1,...,*N* and *N* is the length of the signal, we integrate the signal *u*(*i*) as

$y(k)\equiv\sum_{j=1}^{k} [u(j)-<u>]$ (5)

where *k* = 1,…, *N*, <*u*> is the mean. In the present study, *T_h_*(*i*) is substituted for *u*(*i*). (b) The signal *y*(*k*) is divided into boxes of an equal length *n*. (c) In each box, we fit *y*(*k*) to a first degree polynomial function *y_n_*(*k*), which represents the trend in each box. (d) The signal *y*(*k*) is detrended by subtracting the local trend *y_n_*(*k*) in each box. (e) For a given box size *n*, the root mean square function for the detrended signal $y(k)-y_{n}(k)$ is calculated using

$F(n)\equiv\sqrt{\frac{1}{N'}\sum_{k=1}^{N'} [y(k)-y_{n}(k)]^{2}}$ (6)

where *N’* is the largest integer not greater than *N* / *n*. (f) The procedure shown above is repeated for a broad range of scales (box size *n*) to determine the relationship between *F*(*n*) and the box size *n*.

In the present study, the box sizes are within a range from a minimum of 20 data points to a maximum of *N*/2, where *N* is the length of the time series. In general, the fluctuation *F*(*n*) increases with larger box sizes. A linear relationship in the log-log plot indicates self-similar scaling because fluctuations in smaller boxes are related to fluctuations in larger boxes according to a power law relationship. The slope of the line log *F*(*n*) over log *n* is the fractal scaling exponent *α*, which gives a measure of the “roughness” of the original time series of the stride intervals. Using DFA, a fractal scaling exponent *α* ≈ 0.5 corresponds to rough and unpredictable white noise; *α* ≈ 1.0 corresponds to 1/*f* fluctuation and long-range correlations; and *α* ≈ 1.5 corresponds to a random walk process or Brownian noise [36,37].
